# Hipocrates on Injuries to the Skull—Their Surgical Treatment

**Published:** 1891-09

**Authors:** 


### Hippocrates on Injuries to the Skull—Their Surgical Treatment.

From Adam's recent translation of the genuine works of Hippocrates, issued by Wm. Wood & Co., we quote the conclusions of the author, as summarized by the translator, in this important department of surgery. They are of especial interest just now from the prominence which is being given to brain surgery.

1. Hippocrates recommended the operation of perforating the cranium in cases of simple fractures and contusions, whenever he apprehended that these would be followed by serious consequences, such as inflammation, extravasation of blood or effusion of matter.

2. Hippocrates operated in these cases during the first three days, before any serious symptoms had come on.

3. The objects which he had in view in this class of cases would appear to have been to "slacken the tightness of the skull" and procure the evacuation of extravasated blood.

Hippocrates regarded fracture accompanied with depression and a considerable separation of the bones as being generally less dangerous than severe contusions and simple fractures; as in the former case the brain is less hurt by the vibration of the shock which inflicted the injury, and there is an outlet to any noxious matters which may get congested in the brain.

5. Hippocrates did not operate, as a rule, in cases of depression, not even in cases of comminuted fractures, but left the pieces of bone, in the latter case, to separate by suppuration.

